# Surface data assimilation of chemical compounds over North America and its impact on air quality and Air Quality Health Index (AQHI) forecasts

**DOI:** 10.1007/s11869-017-0485-9

**Published:** 2017-06-10

**Authors:** Alain Robichaud

**Affiliations:** Air Quality Research Division, Environment and Climate Change Canada, 2121 Trans-Canada Highway, Dorval, QC H9P 1J3 Canada

**Keywords:** Data assimilation, Sulfate, Ozone, Particulate matter, Nitrogen dioxide, Vertical correlation length, AQHI

## Abstract

The aim of this paper is to analyze the impact of initializing GEM-MACH, Environment and Climate Change Canada’s air quality (AQ) forecast model, with multi-pollutant surface objective analyses (MPSOA). A series of 48-h air quality forecasts were launched for July 2012 (summer case) and January 2014 (winter case) for ozone, NO_2_, and PM_2.5_. In this setup, the GEM-MACH model (version 1.3.8.2) was initialized with surface analysis increments (from MPSOA) which were projected in the vertical by applying an appropriate fractional weighting in order to obtain 3D analyses in the lower troposphere. Here, we have used a methodology based on sensitivity tests to obtain the optimum vertical correlation length (VCL). Overall, results showed that for PM_2.5_, more specifically for sulfate and crustal materials, AQ forecasts initialized with MPSOA showed a very significant improvement compared to forecasts without data assimilation, which extended beyond 48 h in all seasons. Initializing the model with ozone analyses also had a significant impact but on a shorter time scale than that of PM_2.5_. Finally, assimilation of NO_2_ was found to have much less impact than longer-lived species. The impact of simultaneous assimilation of the three pollutants (PM_2.5_, ozone, and NO_2_) was also examined and found very significant in reducing the total error of the Air Quality Health Index (AQHI) over 48 h and beyond. We suggest that the period over which there is a significant improvement due to assimilation could be an adequate measure of the pollutant atmospheric lifetime.

**Electronic supplementary material:**

The online version of this article (doi:10.1007/s11869-017-0485-9) contains supplementary material, which is available to authorized users.

## Introduction

It is now well known that data assimilation can improve the performance of numerical models (Kalnay 2003). However, assimilation of surface measurements of atmospheric chemicals is particularly challenging due to large representativeness errors associated with topography, significant bias of chemical and meteorological fields adding up together and related to boundary layer processes, proximity to chemical emissions, and different lifetimes of the assimilated pollutants. Near the surface, an accurate numerical forecast is desirable since it improves the ability to predict air quality accurately which better supports decisions to protect the public against adverse effects of unhealthy air quality and also helps to improve epidemiological and etiological studies (Stieb et al. 2008; Crouse et al. 2015). A massive number of research studies have been published describing the impact of poor air quality on human health including eye irritation, asthma, chronic obstructive pulmonary disease (COPD), heart attacks, lung cancer, diabetes, premature death, and damage to the body’s immune, neurological, and reproductive systems (Pope et al. 2002; EEA-WHO 2002; WHO 2003; Sun et al. 2005; Ebtekar 2006; Georgopoulos and Lioy 2006; Pope and Dockery 2006; Reeves 2011; IARC 2013). Although air pollution has diminished over the past decade in North America, a recent report by the American Lung Association (2016) shows that more than one in two people have unhealthy air quality in their communities in the USA (i.e., about 166 million of Americans). In Canada, Robichaud et al. (2016) have produced maps of the Air Quality Health Index (AQHI) which suggest that a majority of Canadians (mostly located in cities in the southern parts of the country) breathe air quality that may pose a risk to their health (i.e. mean AQHI index having values greater than 3) for a significant number of hours on an annual basis (more than 10% of the time). In the USA, ground-level ozone and PM_2.5_ are the primary contributors to poor air quality (EPA 2012). In Canada, the situation is similar and these pollutants are also the main constituents of smog. Together with NO_2_, these pollutants form the basis of the Canadian AQHI which has been designed to take into account the combined impacts on health risk of exposure to a mixture of these pollutants (Stieb et al. 2008). The value of the AQHI and the corresponding health risk message is provided in Table 1. The AQHI index is a risk communication tool, especially targeted at vulnerable populations and computed by using a 3-h moving average of concentrations of ozone, PM_2.5_, and NO_2_. This is the rationale behind the focus of this study on the data assimilation of these three pollutants.

**Table 1:** Air Quality Health Index (AQHI) and its relation to health impact (adapted from http://ec.gc.ca/meteo-weather/default.asp?lang=En&n=8E7198BB-1, last access January 11, 2017)

Health risk	AQHI value	Recommended action
Low	1–3	No action required
Moderate	4–6	Avoid outdoor activities for sensitive population
High	7–10	Avoid outdoor activities (dangerous conditions for sensitive population)
Extreme	>10	Outdoor activities becomes dangerous for the whole population

One of the current weaknesses of air quality models in Canada and elsewhere is that these are not initialized or constrained by chemical observations and hence could contain large uncertainties associated with errors in emissions, boundary conditions, and chemistry parameterization (Pagowski et al. 2010; Robichaud 2010; Moran et al. 2014). Moreover, these models have meteorological inaccuracies associated with wind (speed and direction), atmospheric instability, solar radiation, characteristics of the boundary layer, and precipitation (Reidmiller et al. 2009; Zhang et al. 2012; Bosveld et al. 2014). If models are constrained with chemical observations by initializing with multi-pollutant surface objective analysis (MPSOA), precision and reliability could be improved (Blond et al. 2004; Wu et al. 2008; Tombette et al. 2009; Agudelo et al. 2015). Therefore, data assimilation can correct to a certain extent for model weaknesses as it does in the field of meteorology. Data assimilation provides information at unobserved sites by intelligent physical interpolation and propagation of information from data-rich regions to other regions and also contributes to improve some aspects of observation quality control (Lahoz 2007). Numerous techniques have been developed to improve the performance of short-term air quality forecasts such as bias correction algorithms (Wilczak et al. 2006; Borrego et al. 2011) or chemical data assimilation. Different chemical data assimilation (CDA) methodologies exist including sequential methods such as optimal interpolation (OI), Kalman filtering (KF), extended Kalman filter (EnKF) and variational methods (3D-Var or 4D-Var). Zhang et al. (2012) provide a review of the different techniques. Although the use of OI has diminished in meteorology, it still remains interesting for CDA applications since it does not require high computational resources and yet provides competitive results compared to other methods which are more costly (Wu et al. 2008). Initializing numerical AQ models at regular time intervals with analyses combining models and observations based on OI or other methods can produce accurate air quality forecasts (Blond et al. 2004; Tombette et al. 2009; Messina et al. 2011; Sandu and Chai 2011; Silibello et al. 2014; Agudelo et al. 2015). Many experiments have taken place in Europe and the USA dealing with surface CDA using simple algorithms (Silibello et al. 2014; Augudelo 2015, etc.) or more complex schemes such as variational analysis (Pagowski et al. 2010; Vira and Sofiev 2015). Our results, presented here, are considered the first attempt (i.e. never addressed in the literature) to (1) assimilate surface chemical compounds in Canada in an AQ model and (2) assess the impact of assimilation on the hourly AQ and AQHI forecasts. Objective analyses produced in another context (see description of OI in Robichaud and Ménard 2014; Robichaud et al. 2016) are then assimilated here in an off-line mode and the analysis increments projected in the vertical with different vertical correlation lengths. In the literature, little emphasis seems to be put on the importance of specifying correctly the vertical correlation length or the importance of assimilating individual members of the PM_2.5_ family. In this paper, we show that sensitivity tests of vertical correlation length are required in order to optimize the performance of the CDA algorithm used. Finally, the impact on improving AQHI forecast is also assessed.

## Theory and methods

### Objective analyses of surface pollutants

The impact on the air quality forecast from initializing the GEM-MACH model with surface objective analysis is assessed in this paper. An optimal combination (known as optimal interpolation) of different information leads to a significant improvement of the coverage and accuracy of air pollution patterns and is referred to as chemical objective analysis (COA) (Robichaud and Ménard 2014; Robichaud et al. 2016). More precisely, COA is defined as a combination of observations and short-term forecasts from air quality models which are merged while minimizing an objective criterion. Optimal interpolation (OI) as well as variational methods (3D-Var and 4D-Var) are the foundations of data assimilation and have been extensively utilized in the context of objective analysis over the past decades in meteorology (Kalnay 2003). Optimal interpolation, as used here for air quality objective analysis, is a robust and flexible method to perform data assimilation in air quality and has been shown to give comparable results to the more sophisticated methods such as 3D-Var or even 4D-Var for surface tracer such as ozone (Wu et al. 2008) despite the fact that it uses much less resources than the latter methods. In AQ, pollutants are largely controlled by sources and sinks and boundary conditions as well as atmospheric conditions (Elbern et al. 2010) which all have strong diurnal variations. Therefore, ideally in AQ, data assimilation of hourly observations is desirable and this is why the COAs used here to initialize the GEM-MACH model are produced on an hourly basis. The methodology to produce COAs has been described elsewhere (see Robichaud et al. 2014 and Robichaud et al. 2016). Here, details are given on how the GEM-MACH model is initialized with COA and the impact on the AQ and AQHI forecast performance gained by initializing the model at a specific starting point (usually 00UTC or 12UTC) is also assessed.

**Fig. 1:**
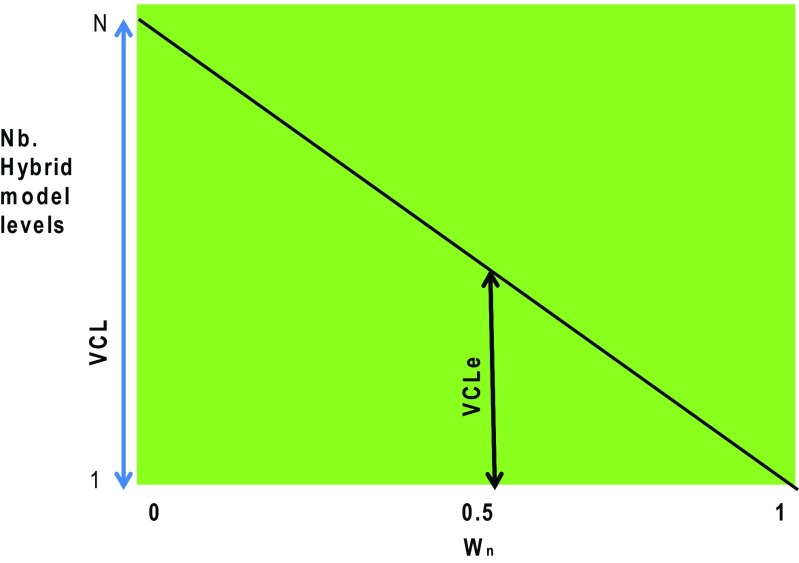
Concept of vertical correlation length (VCL). A decreasing weight *w*_*n*_ in terms of model increasing hybrid levels is given to the surface COA analysis increments. The number of HY levels at which the weight is zero defines VCL, and the number of levels at which the weight falls to half defines the effective VCL_e_

Optimum interpolation (OI) is the technique behind the production of an objective analysis (MPSOA). It consists of a linear combination of the background field and observations optimized by minimizing the error variance using stationary error statistics. The analysis to be assimilated at the surface is obtained as follows (Kalnay 2003):

1 $$ {\boldsymbol{x}}^a={\boldsymbol{x}}^f+\boldsymbol{K}\left({\boldsymbol{y}}^o-{\boldsymbol{Hx}}^f\right) $$

where ***x*** ^***f***^ is the background field obtained from a short-term AQ model forecast, ***H*** is an operator that performs an interpolation from the model grid point space to the observation space (here a bilinear interpolation was used), ***y*** ^***o***^ is a vector that contains all the observations at a given time, and ***K*** is the Kalman gain matrix (of dimension determined by the number of observations and the number of grid points). ***K*** contains the error statistics which minimizes the analysis error and is defined only for the surface model level (see Eqs. 4–6 of Robichaud et al. 2016 for the complete expression for ***K***). Note that modeling error statistics is a challenge in data assimilation (see review of Bannister 2008a,b). The second member of the right-hand side of Eq. 1 refers to the analysis increment. In preparatory work for this study, it was found that initializing only at or near the surface with a given COA does not provide an optimal air quality forecast and projecting the analysis increment in the vertical was found more appropriate. Therefore, a weight (from 0 to 1) is assigned to the analysis increment as a function of the vertical distance so that Eq. 1 is modified as

2 $$ {\boldsymbol{x}}_n^a={\boldsymbol{x}}_n^f+{w_n}^{\ast }{\mathbf{K}}_{\mathbf{s}}\left({\boldsymbol{y}}_{\boldsymbol{s}}^o-\mathbf{H}{\boldsymbol{x}}_{\boldsymbol{s}}^f\right) $$

where the subscript *n* refers to the vertical level of the objective analysis and subscript *s* to the surface level. Figure 1 depicts the decreasing weight (*w*_*n*_) with altitude corresponding to model level *n*. Note that the variables with subscript s are only defined at the surface whereas those with subscript *n* are 3D fields. The number of levels *N* over which *w*_*n*_ is non-zero defines the vertical correlation length (VCL). However, we also define the effective VCL (called VCL_e_ thereafter) as the number of vertical hybrid levels corresponding to where *w*_*n*_ falls to half value (i.e., 0.5). Note that it is the increment (second member of the right-hand-side of Eq. 2), i.e., INCR = *K*_*s*_(*y* ^*o*^_*s*_ − *Hx* ^*f*^_*s*_), which is modified with altitude according to the weight (not ${\boldsymbol{x}}_n^a$). This modified increment is added to the model prediction at the corresponding level from the previous assimilation cycle (according to Eq. 2). The surface COA and its corresponding analysis increment field at the surface (*n* = 1) are obtained from the CMC data archive (for MPSOA) and the associated methodology for COAs described elsewhere (Robichaud and Ménard 2014; Robichaud et al. 2016). Hence, Eq. 2 describes a pseudo-3D analysis since the analysis increment from the surface is applied at all levels *n* through the weighting *w*_*n*_. Note that *w*_1_ (i.e., at the surface, *n* = 1) is equal to 1 and decreases to zero at VCL height (i.e., *n* = *N*). In this paper, all the COA analyses (surface and altitude to a level *N*) are used to initialize a series of 48-h AQ forecasts (GEM-MACH model). This air quality model is part of the Canadian Air Quality Regional Deterministic Prediction System (AQRDPS) with a spatial resolution of 10 km (Moran et al. 2012). The objective analysis exploits air quality surface observations from the US AIRNow program (Aerometric Information Retrieval Now), as well as Canadian observations measured in real time by provinces and territories (and some municipalities) (see Robichaud and Ménard 2014 or Robichaud et al. 2016 for more details).

### Surface observations

**Fig. 2:**
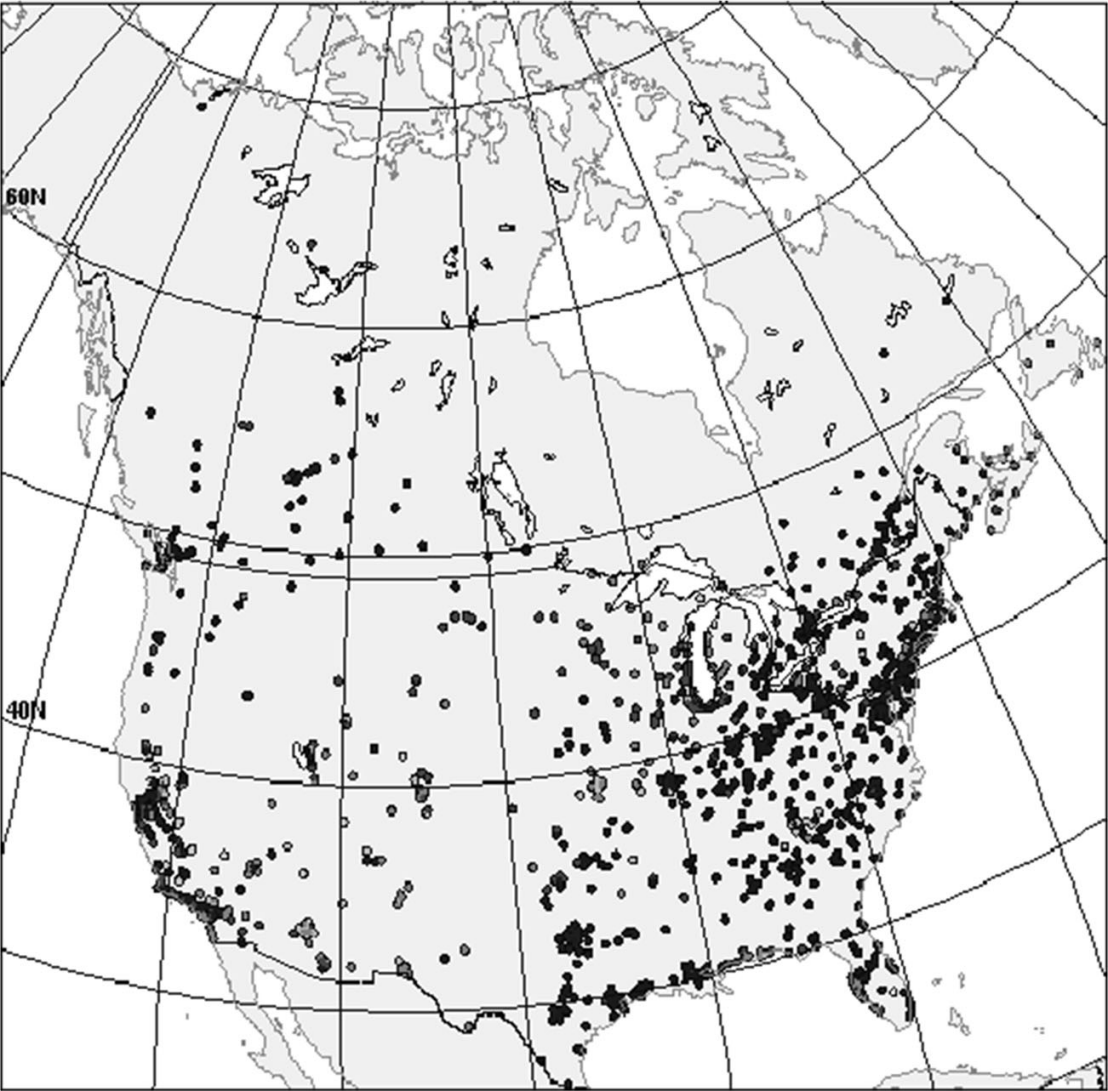
Observation sites used in data assimilated and validation include AIRNow surface sites (US/EPA database) and Canadian stations

The surface observations utilized in the MPSOA are received at CMC and are rigorously quality-assured (for details, see supplementary material S1B of Robichaud et al. 2016). How well observations represent the pollution concentration in a given region depends largely on local emission sources, topography and meteorology, boundary-layer characteristics, and the lifetime of the pollutant of interest. Observation errors are of three kinds: (1) systematic, (2) random, and (3) representativeness (Lahoz et al. 2007). The spatial representativeness of a monitoring station should depend in some aspect on surrounding land use (as discussed in Silibello et al. 2014 and Bédard et al. 2015). Figure 2 shows the location of the monitoring sites used to produce MPSOA in the RDAQA system and also used to evaluate the accuracy of the GEM-MACH air quality forecast model in both assimilation and non-assimilation mode. The density of sites is high over eastern USA and California (Western USA) and the Gulf states and becomes lower elsewhere in the USA and southern Canada with little density in northern Canada. For PM_2.5_, the number of sites is about half that of ozone although the geographical distribution of sites is fairly similar. NO_2_ observations are numerous only in southern Canada (except for Alberta which is well covered by monitoring stations) and scattered in USA (see Robichaud et al. 2016 for more details). Adequate observation coverage is a challenge in Canada due to the large extent of uninhabited areas. Typical measurement techniques for different pollutants are described elsewhere (see Robichaud et al. 2016, their Table 2).

**Table 2:** Description of the different assimilation experiments performed

• Exp. (A): assimilate PM_2.5_ sub-species (sulfate, organic carbon, nitrates, ammonium, crustal material, elemental and primary carbon) one by one individually (non-cumulative impact on AQ forecast) and note the impact of each sub-species in improving PM_2.5_ 24-h forecast (period July 2012)
• Exp (B): same as (A) but assimilate all PM_2.5_ (with cumulative impact) over a 48-h forecast
• Exp (C): add-up assimilation of ozone and nitrogen dioxide (period July 2012)
• Exp (D) D1: winter case for PM_2.5_, D2: winter case for O_3_ and NO_2_ (January 2014)

### Air quality model

The air quality model used in this study is the GEM-MACH model (version 1.3.8.2 for chemistry and 5.0.4.4 for physics) which is a limited area air quality operational model developed at Environment and Climate Change Canada. GEM-MACH is run online **(**chemistry online with meteorology), and its boundary is driven by the global meteorological model GEM (Côté et al. 1998a, b; Mailhot et al. 1998; Moran et al. 2014). The domain for the objective analysis is the same as the model domain and essentially covers North America with a spatial resolution of 10 km as well.

### Methodology to obtain the optimum vertical correlation (length)

The vertical correlation length (VCL) corresponds to the number of vertical levels *N* over which the analysis increments are projected. We perform sensitivity tests to determine the profile *w*_*n*_ in Fig. 1 from which the optimum value of VCL can be obtained (i.e. when it reduces the forecast error to a minimum while maximizing some metrics such as the frequency of being correct within a factor 2). Computation of different metrics is described in the “Validation” section. In the literature, little reference is made to the sensitivity of the VCL in data assimilation studies over large regions so there is no way to know if the vertical correlation is optimum. Nevertheless, Silibello et al. (2014) found local values of optimum VCL (which minimize RMSE performance index) but no details were given on how to extrapolate these values to larger regions or to other chemical compounds. Here, global values of optimum VCL are obtained in terms of model hybrid levels which could be converted to approximate pressure levels if needed to facilitate the implementation to a different model.

### Methodology to project the surface analysis increments in the vertical

The following procedure is adopted to project the analysis increment in the vertical. The basic equation which describes the analysis increment at a model hybrid level HY for a given pollutant tracer *T*_*j*_ is given as

3 $$ \mathrm{INCR}\left(\mathrm{HY},{T}_j\right)=\mathrm{INCR}{\left(\mathrm{sfc}\right)}^{\ast}\mathrm{a}{\left(\mathrm{HY}\right)}^{\ast}\mathrm{pratio}\left({T}_j\right) $$

where INCR (i.e. second member to the right-hand side of Eq. 2) is the weighted 3D analysis increment for a given chemical tracer *T*_*j*_ at a given hybrid level *n* = HY, INCR(sfc), the surface analysis increment, a(HY), the vertical weight profile which depends on the hybrid level, and pratio(*T*_*j*_), the partitioning ratio for sub-species. Note that the latter is unity for ozone and NO_2_ but not for PM_2.5_. Note also that the vertical coordinate used here (i.e. *n* = HY) is a model terrain following coordinate. It is believed that it is more natural to express VCL in terms of the number of HY levels rather than pressure levels or standard altitude levels which have discontinuities or non-existing values (e.g. pressure level below ground). Moreover, it simplifies the interpretation of results for the impact on assimilation in relation to VCL. For PM_2.5_, the partitioning ratio depends on the ratio of a sub-species (sulfate, nitrate, ammonium, organic carbon, primary carbon, elemental carbon, crustal material) over the total mass of assimilated PM_2.5_. The partitioning ratio could be obtained either from the previous model outputs every 12 h or by some kind of monthly climatology (monthly values of partitioning ratio have been computed for July 2012 and January 2014 for PM_2.5_ in order to obtain this climatology). For ozone and nitrogen dioxide, pratio equals 1 (in Eq. 3) since there are no sub-species and therefore no partitioning required for these two pollutants. The vertical shape of the weighting ratio a(HY) was chosen as a linear decrease (in terms of the model hybrid vertical levels) from surface to the optimum vertical correlation length. Note that although the decrease of the weights is linear in terms of model hybrid levels (i.e. Fig. 1), it turns out to be exponential in terms of altitude. As an example, in summer, say VCL = 20HY, then at the first model level near the surface, the weight equals 1. At the fifth hybrid model levels (altitude of about 600 m) the weight equals 0.75, at the 10th level (∼1250 m) it is 0.5, at the 15th level (∼2800 m) it is 0.25, and finally vanishes at the 20th level (altitude of 4600 m approximately). Other profiles such as stepwise or exponential were tried (instead of linear as in Fig. 1) but found to give no improvement of the results. The optimum vertical correlation length (optimum number of levels) is obtained through sensitivity tests (vertical correlation length which minimizes predefined metrics such as the unbiased root mean square error, the mean and absolute mean bias, and the frequency of being correct within a factor of 2). Once VCL is obtained, we compute the analysis increment by Eq. 3 and re-initialize the model using Eq. 2 and perform a 48-h forecast at two times 00UTC and 12UTC. Validation metrics are described below. Note that the vertical projection of the analysis increment is a different procedure than that projecting the analysis itself. The former methodology as expressed by Eq. 3 does not appear in the previous literature.

### Validation

Four metrics are used to establish the performance of MPSOA and are defined in Appendix 1: (1) mean bias (average O-P or Observation minus Prediction and O-A, i.e. Observation minus Analysis), (2) mean absolute bias, (3) standard deviation of O-P and O-A to evaluate random error (i.e. which is equivalent to unbiased RMSE for large *N*), and (4) frequency of being correct within a factor 2 (FC2) to assess reliability. Note that the metric FC2 is a more robust measure of the reliability since it is not sensitive to “outliers” or “compensating errors” (Chang and Hanna 2004). The best performance is obtained when the total error (TE) is minimum and when FC2 is maximum (see definition in Appendix 1). Note also that OmP and OmA will be used thereafter instead of O-P and O-A. Finally, since observations of PM_2.5_ contain sea-salt, while sea-salt in the model is separate from PM_2.5_, a correction to remove sea-salt from observations has been made based on the average model ratio of sea-salt to total PM_2.5_. Note that all hours in this study are expressed in Greenwich international time (UTC, i.e. 00Z, 12Z, etc.).

## Results

In a previous study (Robichaud and Ménard 2014), the *horizontal* correlation length was shown to be a quite sensitive parameter for the accuracy of the COAs. During the course of the work presented here, it was found in the various numerical experiments that the *vertical* correlation length could also be a quite sensitive parameter for the 3D data assimilation of PM_2.5_ and ozone but much less for NO_2_. To demonstrate this, a series of AQ 24- or 48-h forecasts using the GEM-MACH model initialized by MPSOA were launched with different VCL values. After the optimum VCL was established, additional experiments were launched (see description in Table 2). Below we examine two cases: July 2012, representative of the summer season, and January 2014, representative of the winter season, for three species: PM_2.5_, O_3_, and NO_2_.

### Sensitivity tests for the vertical correlation length

**Fig. 3:**
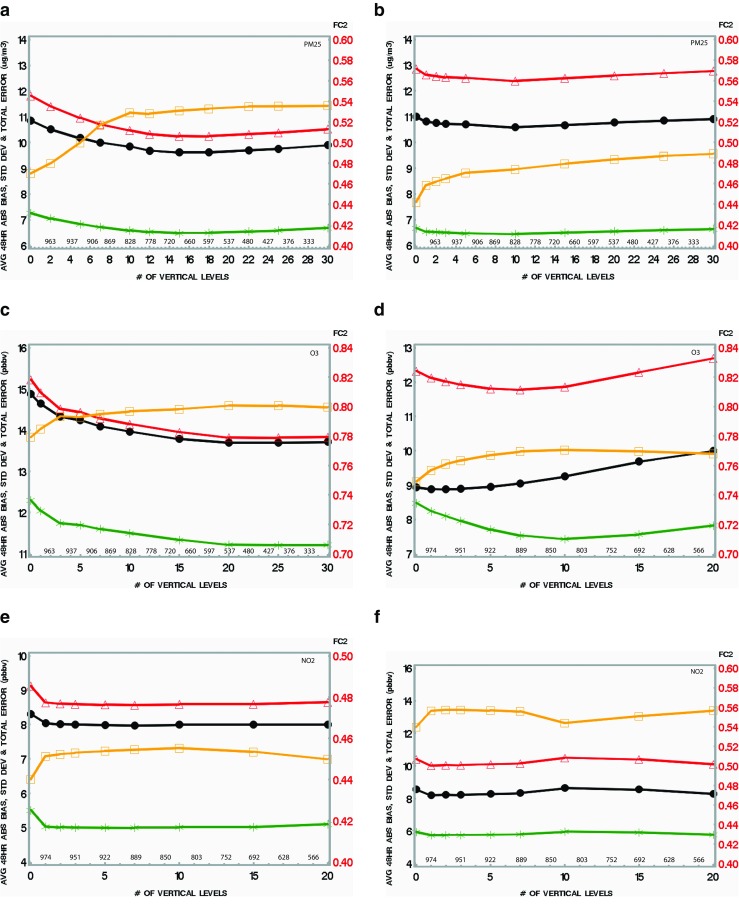
Sensitivity tests for the vertical correlation length: July and January case for PM_2.5_ (**a**, **b**), ozone (**c**, **d**), and NO_2_ (**e**, **f**). *Green curves* stand for the mean absolute bias, *black* for the standard deviation of OmP (random error), and *red curves* for the total error (combining bias and standard deviation errors, see Appendix 1). Finally, *orange curves* describe the sensitivity of the FC2 metric. Approximate values of pressure (in millibars) are indicated on top of the *abscissa* (color figure online)

The optimum correlation length is the one which will optimize the performance of the model using the validation metrics described in Appendix 1. Model values were interpolated at the observation sites, and a mean 24-h performance was then computed for each site (a geographical map of the observation sites used to compute model versus residuals is given in Fig. 2). Starting with PM_2.5_ sensitivity tests, Fig. 3a shows that, in summer, the minimum error (mean absolute bias, standard deviation of OmP, and total error of OmP) all occur when the vertical correlation length is around 15 hybrid levels (pressure level P ∼ 715 mb). Concerning the FC2 metric (orange curve), it reaches a maximum (around 0.54) at about 15 levels as well. We therefore conclude that VCL = 15 hybrid levels is the appropriate vertical correlation length for PM_2.5_ in summer. For ozone and NO_2_ (Fig. 3c, e, respectively), the different metrics are optimum for 20 levels (P ∼ 560 mb) and beyond for ozone and 4–10 levels (P ∼ 940–840 mb) for NO_2_. For the case of the winter season (Fig. 3b, d, f), a similar method was used to obtain optimum VCL. However, it is found that the optimum VCL is smaller and not as sharply defined compared to the summer case. The optimum VCL for the winter case is about 10 levels for PM_2.5_ (Fig. 3b), 5–10 levels (P ∼ 925–840 mb) for ozone (Fig. 3d), depending on the metric, and 1–6 levels (P ∼ 960–900 mb) for NO_2_ (Fig. 3f). Note that for the winter case (January 2014), optimum VCL for PM_2.5_ is in the range 5–15 vertical levels depending on which metric we consider, although the FC2 metric keeps growing slightly beyond that level 15 for reasons that are unclear (Fig. 3b). Nevertheless, it is suggested to adopt the optimum VCL as 10 levels since all other metrics point towards optimality for that value. For ozone (Fig. 3d), the optimum VCL also varies depending on which metrics we are examining: the absolute bias of OmP is lowest for VCL = 10 levels (green curve) and FC2 is also highest for that VCL value (orange curve). However, the total error of OmP is lowest for about seven HY levels (red curve) and the standard deviation (black curve) is lowest for even lower values (two to five first HY levels). We suggest here to choose optimum VCL corresponding to 10 HY levels as well since two metrics (mean absolute bias and FC2) are also optimum for that value of VCL. For NO_2_ during January 2014 (winter case, Fig. 3f), optimality was found at very low altitude (i.e. from one to six vertical levels). The result that VCL is smaller in winter is consistent with the fact that the boundary layer height is lower in winter than it is in summer for a given location. Note that for NO_2_, optimum VCL was found at an altitude much lower than the usual depth of the boundary layer and smaller than the VCL for ozone and PM_2.5_ in both seasons. We suggest that due to the shorter lifetime of NO_2_, the signal of surface assimilation does not reach higher altitude and therefore optimum VCL is shorter than ozone and PM_2.5_. A lack of sensitivity to assimilation of NO_2_ was found in this study, and this can be seen in Fig. 3e, f where changing the vertical correlation length brings very little change in the performance as measured by numerous independent metrics for both seasons. This contrasts with the sensitivity for ozone and PM_2.5_ which are both more pronounced as shown in Fig. 3, but again, this is related to the chemical species characteristics. Note that the results for NO_2_ obtained above are consistent with observations of vertical profiles made in Germany (Veitel 2002).

Table 3 contains a summary of the results found for optimum VCL for each pollutant and each season in relation to the structure of the atmospheric boundary layer (ABL). Intuitively, optimal vertical correlation length should correspond to the top of the ABL (corresponding in summer to around 7–10 model hybrid levels) for long-lived tracers since within that layer, these tracers are well mixed. This differs from NO_2_ which lies within the surface layer or little above but rarely gets to the whole ABL (as shown by model outputs without assimilation, figure not shown). The effective optimum VCL (i.e., VCL_e_), i.e., defined here as the vertical correlation length at half-length, matches better with our intuitive notion of the depth of the mixing layer (see last column of Table 3 for the correspondence with altitude). Based on the above experiments, it was found that the vertical correlation length is indeed a very sensitive parameter (for PM_2.5_ and ozone) and finding the optimum VCL should be recognized as a mandatory step for sound surface assimilation experiments. Theoretical derivation of an optimal VCL was avoided here since many hypotheses would be required concerning the surface layer, boundary layer, atmospheric lifetime of compounds, and representativeness errors. Such information is generally lacking so we have instead chosen to conduct sensitivity tests using real-world data. Note that the experiments described in the remainder of this paper all used the optimum VCL found in this section.

**Table 3:** Summary of results of sensitivity tests for VCL (ABL stands for atmospheric boundary layer)

Species (season)	No. of vertical levels (optimum VCL)	Effective VCL (VCL_e_) (nb. Hybrid levels)	Approximate altitude of VCL_e_
PM_2.5_			
(Summer)	15	7.5	800 m (near top of ABL)
(Winter)	10	5	400 m
O_3_			
(Summer)	20	10	1.2 km (top of ABL)
(Winter)	5–10	2.5–5	100–300 m (above surface layer)
NO_2_			
(Summer)	4–10	2–5	100–400 m
(Winter)	2–6	1–3	<200 m (mostly surface layer)

Reference to ABL structure is from Stull (1988)

### Impact of assimilating PM_2.5_ sub-species individually on the AQ forecast (Exp. A)

Once the optimum VCL was found, the impact of assimilation was evaluated using various experiments. The assimilation of PM_2.5_ involves a partitioning for sub-species since the model requires initialization with individual sub-species, not with the PM_2.5_ aggregate analysis made from observations. It is possible to assimilate every sub-species individually in order to find which of the sub-species has the most impact in improving the mean PM_2.5_ air quality forecast. In the literature, the assimilation of PM_2.5_ which has been performed is usually related to the fine aerosol total mass (e.g. Pagowski et al. 2010). In here, the impact of individual sub-species is examined in order to identify which member of the PM_2.5_ family has the most impact in improving the forecast. Note that unlike in the model, the partitioning of PM_2.5_ observations is not a priori available since only the mass of the whole family of fine particles is routinely measured and no monitoring takes place on a routine hourly basis at the level of the sub-species at the present time in Canada. The seven sub-species which are part of the assimilation process are sulfate (SU), crustal material (CM), nitrates (NI), ammonium (AM), organic carbon (OC), elemental carbon (EC), and primary carbon (PC). Partitioning was done by using a GEM-MACH model climatology (monthly average values) of ratio of sub-species over total PM_2.5_ (excluding sea salt) for either summer or winter (i.e. using model runs without assimilation performed for July 2012 and January 2014). Note that a model climatology was used, since it was found that a climatology versus on-the-fly ratio from the model was less noisy and consequently gave slightly better results. This model climatology was first compared to the ratio of sulfate, nitrate, and ammonium to total PM_2.5_ mass, respectively, using data from CAPMON, CASTNET, and IMPROVE networks available in North America. The comparison was found reasonable for both seasons (results not shown here).

Table 4 shows the performance averaged over the first 24-h forecast period for each species assimilated individually compared to the performance of the model without assimilation (NO ASSIM). Assimilation of each individual sub-species improves the performance (higher FC2 and lower absolute bias, std. dev., and total error) as compared to the model without assimilation as expected. However, assimilation of sulfate and crustal material shows the most important improvement as revealed by the total error reduction (bottom entry of the table in %) compared to the case of no assimilation. A weaker performance occurs for nitrates and ammonium followed by primary and elemental carbon. The likely explanation for better performance for sulfates and crustal materials is linked with a longer atmospheric lifetime than the remaining sub-species. Longer lifetime means that the information can be transported over larger distances and improve scores over larger regions.

**Table 4:** Performance of individual assimilation of sub-species (units are in µg/m^3﻿^ except for FC2 which is a fraction)

Metric/species	SU	NI	AM	CM	OC	EC	PC	NO ASSIM
Abs. OmP	5.79	6.79	6.66	6.50	6.55	6.73	6.59	7.15
Std. dev. OmP	8.46	9.96	9.70	9.64	9.74	9.90	9.69	10.77
FC2	0.561	0.479	0.480	0.516	0.519	0.488	0.491	0.484
Total error	10.25	12.05	11.77	11.63	11.74	11.97	11.72	12.93
(% total error reduction)	(25%)	(6.4%)	(8.7%)	(12.0%)	(11.3%)	(7.6%)	(9.8%)	–

The mean percent error reduction is relative to the case of a model run without assimilation (NO ASSIM)

SU sulfate, NI nitrates, AM ammonium, CM crustal material, OC organic carbon, EC elemental carbon, PC primary carbon

### Impact of assimilating fine particle (PM_2.5_) sub-species altogether (Exp. B and D1)

**Fig. 4:**
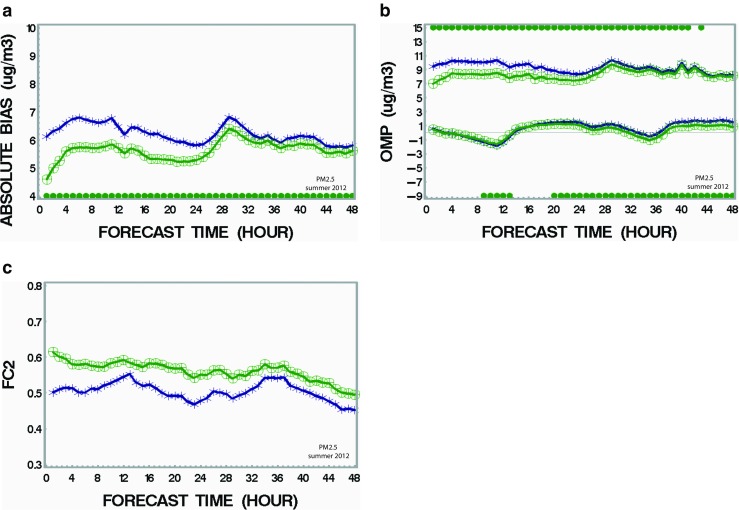
Impact of assimilation of PM_2.5_ on the 48-h air quality forecasts (summer case) on **a** mean absolute bias, **b** standard deviation of OmP and mean bias, and **c** FC2. *Green curves* are for the run with assimilation. *Navy curves* represent the base case (no assimilation). For standard deviation and bias error, *green dots* at the *top* or *bottom* indicates respectively difference between the two runs which has statistical significance (*p* < 0.05). Time is expressed in terms of number of hours after forecast launch (color figure online)

We also examined the impact of assimilating all sub-species of PM_2.5_ together using the optimum values of VCL found in the previous section (Table 3). The hourly performance of these optimal runs is examined up to a 48-h forecast period and compared with the run without assimilation. Figure 4 shows the results for the following metrics: absolute bias, standard deviation of OmP, and FC2. Only the results of the model runs starting at 00Z are shown here since runs at 12Z have roughly similar patterns and therefore do not bring new information. Figure 4a shows that the absolute bias of the data assimilation run (green curve) is significantly lower than the run without assimilation (navy curve) for the summer case (July 2012). Note that a statistical significance with *p* < 0.05 is indicated by a green dot at the bottom of the figure as obtained from a statistical sign rank test (T-test is not used here since the distribution is not normal for mean absolute bias). Reduction of the standard deviation of OmP (top curves of Fig. 4b) for the assimilation case (green curve) is also noted for a period up to about 40 h for the summer case. For the mean biases (bottom curves of Fig. 4b), note very little change for the assimilation case (green curve) as compared with the case without assimilation (navy curves) although the differences are significant for a large window of the 48 h (mainly because *N* is large, i.e. ∼4000). The fact that both curves almost superimpose suggests that assimilation does not resolve the problem of mean bias (compensating errors). Note that an F-test is used to compare standard deviation curves (top curves in Fig. 4b) whereas a *T-* test determines if significant differences exist between mean bias curves (bottom curves in Fig. 4b). The last metric, FC2 (frequency of correct forecast within a factor 2 as compared to observations), shows the largest improvement of all metrics for the assimilated case (green curves) in summer as compared to the case without assimilation (navy curves). Note that the signature of diurnal cycles in the performance curves is present for all metrics discussed in Fig. 4. Note also that it was found that assimilating only sub-species SU and CM would contribute almost totally to the reduction of the error and that adding up other sub-species (i.e. NI, AM, OC, PC, EC) in the assimilation cycle would almost produce no further improvement. Again, it is known that SU and CM have a longer lifetime than the other sub-species considered here and the former sub-species likely mix within the whole boundary layer and contribute the most to the success of PM_2.5_ assimilation since the information assimilated from observations gets transported over a large part of the domain. The winter case (January 2014) shows similar results than the summer case but with less improvement compared to the basic case (no assimilation) for all metrics; see Supplementary material, Fig. S1A (absolute bias), Fig. S1B (mean and standard deviation of OmP), and Fig. S1C (FC2). The better score for assimilation in summer as compared to winter is likely due to a longer lifetime of PM_2.5_ and a deeper boundary layer during the warm season as mentioned above. But, in any cases, in both seasons, the runs with assimilation (green curves) are clearly showing more accuracy (less bias and random error) as well as more reliability (i.e. higher FC2) than the model free run (base case: no assimilation).

### Impact of assimilating gas concentrations (ozone and nitrogen dioxide: Exp. C and D2)

**Fig. 5:**
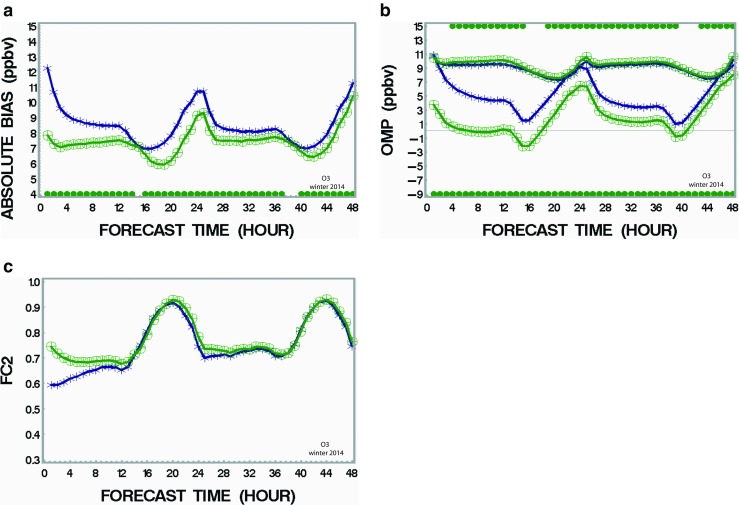
Impact of assimilation of ozone on the 48-h air quality forecasts (winter case) on **a** mean absolute bias, **b** standard deviation of OmP and mean bias, and **c** FC2. *Green curves* are for the run with assimilation. *Navy curves* represent the base case (no assimilation). *Green dots* at the *top* or *bottom* indicate difference between the two runs which has statistical significance (*p* < 0.05). Time is expressed in terms of number of hours after forecast launch (color figure online)

In a similar way, model runs were made by initializing at 00Z and 12Z with objective analyses of ozone and NO_2_ assimilated together. The performance of these runs is compared with the case of no assimilation in Fig. 5. Mean absolute bias over the whole North America during winter (green curves in Fig. 5a) is significantly diminished when ozone and NO_2_ assimilation occurs when compared to model runs without assimilation (navy curve). For the summer case, Fig. S2A (Supplementary material) shows similar results. Note the presence of strong diurnal cycles of the absolute bias for both model runs (with and without assimilation) which is likely due to the diurnal photochemistry cycle for ozone and diurnal boundary layer depth variations. Smaller differences (sometimes not even statistically significant according to the sign rank test) between the assimilated and non-assimilated runs tend to appear in the morning (forecast hour 14–18 UTC and 38–42 UTC) for both summer and winter cases. The reason for this could be due to (1) stronger representativeness errors in the morning due to an ill-defined boundary layer at that time of day and (2) rapidly changing morning photochemistry for ozone and NO_2_ (mostly in summer). Both would reduce the performance of the assimilation impact because the rate of change of ozone is faster than the assimilation frequency (every 12 h here). When the boundary layer and the photochemistry both stabilize, positive impact of assimilation shows up again. Figure 5b shows the standard deviation (OmP, top curves) and mean biases (bottom curves) for the summer case. A slightly better performance (but statistically significant as indicated by green dots at the top for most of the 48-h period in winter) is noted for the assimilated case versus the non-assimilated case. The mean bias (bottom curves of Fig. 5b) is strongly reduced in the case of assimilation (green curve) as compared to the model run (navy curve) without assimilation (the differences are statistically significant over the full 48-h period). Corresponding results for the summer season are shown in Supplementary material (Fig. S2A and Fig. S2B). Some similarity is found for the absolute bias (similar strong diurnal cycles and hourly performance). However, the mean bias is only slightly improved in summer as compared to the winter case. Note that the impact of assimilation of ozone and NO_2_ on ozone concentration holds even beyond the 48-h period during the winter season (which is less obvious for the summer case). It is believed that there is less presence of compensating or cumulative errors during winter due to less photochemistry during that season in North America as compared to the summer case. The last metric, FC2, shows only small differences between the assimilated versus non-assimilated cases (some important differences are noted in winter only for the first 12 h, Fig. 5c). A similar result is noted for the summer case (Fig. S2C). It is suggested that for ozone (contrary to the case of PM_2.5_), this metric is not very sensitive and perhaps inappropriate to estimate the performance for this compound. This is explained by the fact that ozone is usually well forecast within a factor of 2 by air quality models which is not the case for PM_2.5_. Comparing Fig. 4c (FC2 in the range 0.45–0.60) for PM_2.5_ with Fig. 5c (FC2 in the range 0.6–0.95) for ozone clearly supports the latter statement (i.e. assimilation of ozone does not improve substantially the metric FC2).

Only the runs initialized at 00Z were shown above since the run initialized at 12Z did not bring new information and had roughly similar patterns than that of the 00Z run. In the above experiments, both ozone and NO_2_ were assimilated as it was found that assimilating individually the two compounds would not improve the AQHI forecast (e.g. a deterioration of NO_2_ performance is noted if only ozone is assimilated, figure not shown). Finally, when both ozone and NO_2_ are assimilated (model run initialized by objective analysis), the impact on NO_2_ model performance was found to be very limited and mostly non-statistically significant in both seasons (figures not shown). The likely explanation is that since NO_2_ lifetime is much shorter than ozone and PM_2.5_, the impact of assimilation is expected to also last for a much shorter period and therefore not transported over large regions and consequently having only local impacts. Not surprisingly, the sensitivity of VCL for NO_2_ is also quite small (Fig. 3e, f) compared to PM_2.5_ and ozone. Moreover, the number of stations reporting NO_2_ is much less than that for other pollutants limiting its utility in assimilation (especially over the US territory).

### Impact of assimilation on the Air Quality Health Index forecast

**Fig. 6:**
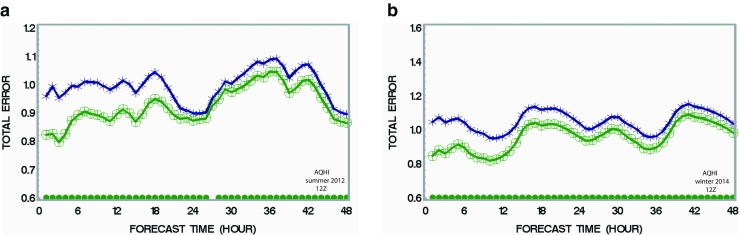
Impact of combining assimilation of the three pollutants (PM_2.5_, ozone, and NO_2_) on the AQHI performance (12Z case). Total error of AQHI for **a** July 2012 and **b** January 2014. *Green dots* at the *bottom* indicates difference between the two runs which has statistical significance (*p* < 0.05) (color figure online)

The Air Quality Health Index (AQHI) was developed in Canada by Stieb et al. (2008) to communicate the risk to sensitive individuals due to short-term exposure to air pollution, namely, from three pollutants and their interactions: ozone, PM_2.5_, and nitrogen dioxide. The formula used to compute and map AQHI is given as

4 $$ \mathrm{AQHI}={\frac{10}{10.4}}^{\ast}\left[{100}^{\ast}\left(\left( \exp \left({0.000871}^{\ast }{\mathrm{NO}}_2\right)-1\right)+\left( \exp \left({0.000537}^{\ast }{\mathrm{O}}_3\right)-1\right)+\left( \exp \left({0.000487}^{\ast }{\mathrm{PM}}_{2.5}\right.\right)\left( \exp \left({0.000487}^{\ast }{\mathrm{PM}}_{2.5}\right.\right)\left.-1\right)\right)\right] $$

where NO_2_, O_3_, and PM_2.5_ are the model forecast values initialized by COA. Since we have made runs of assimilation for the three pollutants (in order to compute a model-grid value of AQHI, Eq. 4), it is interesting to compare the impact of simultaneous assimilation of the three pollutants on the AQHI 48-h forecasts (computed according to Eq. 4 for both assimilation run and base case). According to Eq. 4, more accurate inputs (concentrations of the three pollutants based on data assimilation) should improve the accuracy of AQHI. However, successful individual assimilation of ozone and PM_2.5_ or NO_2_ does not guarantee a better AQHI forecast due to possible adverse synergy (i.e., cross-biases, cumulative or compensating errors, etc.). Figure 6a, b shows that, in summer and winter, respectively, for the 12Z run, the total error (a metric which combines absolute bias and standard deviation of OmP, see Appendix 1) is reduced by up to 30% (*p* < 0.05) as compared to the run without assimilation. The impact on AQHI reveals the combined impact of the three pollutants under study and turns out to be overall slightly stronger during winter than summer but lasts beyond 48 h for both summer and winter (as indicated by the significance test, i.e. green dots at the bottom of the figures) These results for the impact on AQHI are not a priori trivial because the different biases of the three pollutants do not necessarily cancel or add up (i.e. they could have resulted in a negative or positive synergy to the total bias but they did not, at least, in an obvious way). The case for the 00Z run is similar and shown only in Supplementary materials (Fig. S3A for summer and Fig. S3B in winter). The fact that both 00Z and 12Z runs roughly show the same result suggests that the synergy between three different pollutants (considering different diurnal cycles) is not strong. In summary, we conclude that, overall, the assimilation significantly improves the AQHI forecast for most of the 48-h forecast and beyond but more in winter than in summer.

### Mapping geographical differences of the impact of assimilation

**Fig. 7:**
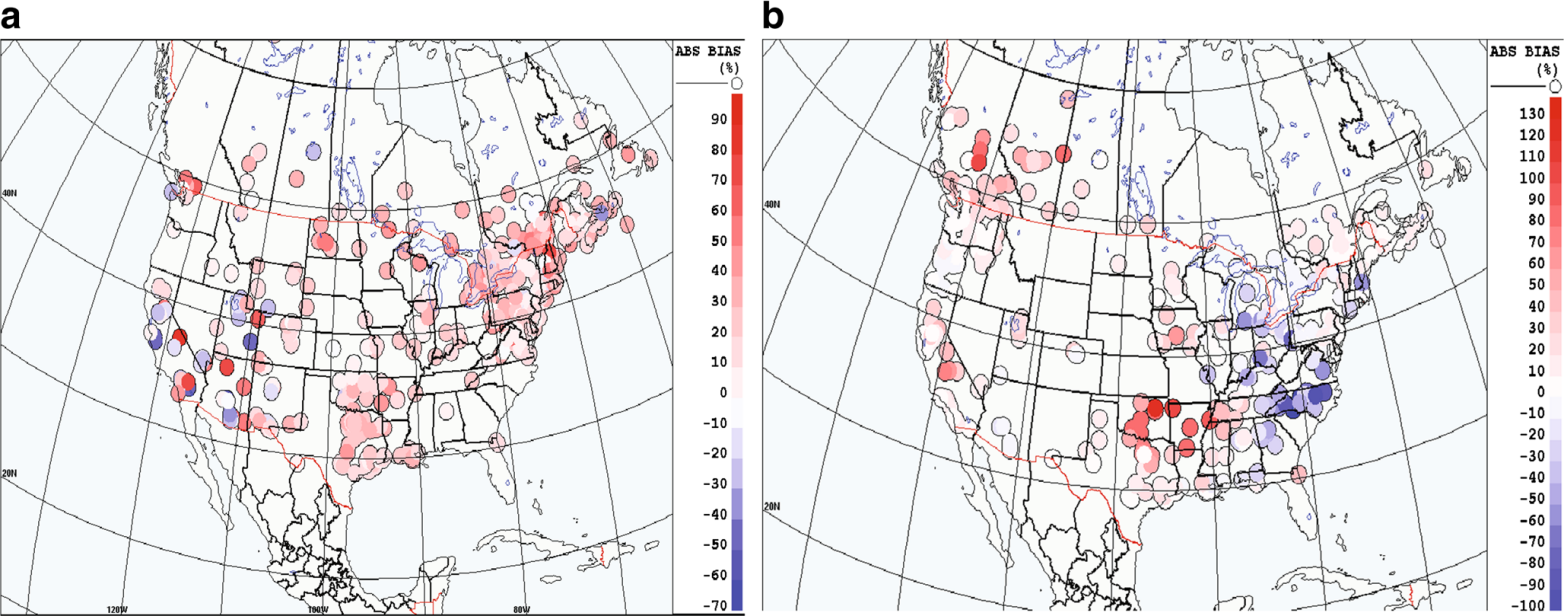
Percentage improvement of mean absolute bias (based on 24 h forecast in North America) (summer case, i.e. July 2012). **a** PM_2.5_. **b** Ozone

It is interesting to examine geographical differences for some metrics in order to evaluate the spatial impact of assimilation and to detect potential problems at different spatial scales. A mapping of the percentage of improvement of scores for the absolute bias of OmP appears in Fig. 7 for both PM_2.5_ and ozone and for the summer case. The mapping used a mean 24-h forecast computed over a whole month (July 2012) so the differences between the assimilated run versus base case are considered highly significant (*N* ∼ 4000). In Fig. 7, reduction of the absolute bias for the assimilation run as compared to the base case (run with no assimilation) is noted for most of the locations (i.e. sites with any grade of red means that performance is improving with assimilation). However, for some locations, for ozone, there is a degradation of performance (corresponding to any grade of blue on Fig. 7b located in eastern USA). Some sites also show no or little change (white color). The winter case (see Supplementary material, Fig. S4) shows similar results except for few sites which show degradation of performance for ozone mostly over Western USA (perhaps due to mountain-valley or sea-breeze misrepresentation from the model and assimilation algorithm due to non-representative VCL at these sites). For PM_2.5_ in winter (Fig. S4a), a widespread improvement of the absolute bias (i.e. reduction from 10 to 100%) occurs in North America. The reason for the degraded performance at certain sites is unknown, but this information is nonetheless useful since it can help to track any possible issues occurring relative to these regions (e.g. model emissions, boundary layer mismatch between real and model topography, instrument or quality control assimilation algorithm, fast photochemistry time scales). The sites showing systematic degradation of performance should then be flagged or possibly removed from the list of assimilated sites in future versions or they could be further analyzed to build better assimilation quality controls because they could be possible outliers. Future algorithms for assimilation should perhaps determine VCL according to local or regional characteristics and be dynamical and not static and defined globally as is the case here.

### Robustness of results

The results are robust and seem to be model independent. In fact, similar results for vertical correlation length for ozone were obtained with the CHRONOS model (in use for operational forecasting from 2001 to 2009 in Canada, see Ménard and Robichaud 2005). Moreover, results found with other versions of GEM-MACH (e.g. using the version with 15-km resolution; unpublished results) were also similar to the one found here (v1.3.8.2, 10-km resolution) and support the notion that the optimum vertical correlation length is linked with the chemical and physical characteristics of the compounds and not with model artifacts. Note that tests using the model output value of the boundary layer height in place of optimum VCL derived here show a slight deterioration of results or in any case no improvement compared to the methodology described here (using sensitivity tests for VCL). Similarly, scaling VCL with boundary-layer parameters such as the friction velocity or bulk Richardson number did not give better results than that presented in this study.

## Summary and conclusion

Improving air quality forecasts through data assimilation is an important step towards a more efficient total environmental risk monitoring system. Models are generally characterized by known deficiencies for prediction of many pollutants whereas measurement systems suffer from representativeness problems and lack of sufficient coverage and, thus, are often best suited for providing local air quality information. This paper is the continuation of a previous scientific project where multiple pollutant surface objective analyses (MPSOA) were prepared using optimal interpolation techniques combining air quality model (GEM-MACH) and AIRNow database supplemented by Canadian surface observations (Robichaud and Ménard 2014; Robichaud et al. 2016). These MPSOA have been made available for operations since 2013 at the Canadian Meteorological Centre (CMC) as part of a near real-time operational product. In the current study, a series of 48-h air quality forecasts for ozone, NO_2_, and PM_2.5_ were initialized by these archived MPSOA. In the study presented here, we focused on these three pollutants since they are the most significant for AQ forecasting. Moreover, they are the inputs to the Air Quality Health Index in Canada (AQHI developed in by Health Canada, Stieb et al. 2008). The aim of this paper was (1) to present the impact of assimilating surface observations in North America using the MPSOA and air quality (AQ) forecast from GEM-MACH model, a state-of-the-art model used in Canada for AQ, and (2) to relate the success of assimilation to optimum VCL and the atmospheric lifetime of the chemical compound. Results show that assimilation of PM_2.5_ (ozone) has a significant impact which is stronger in summer (winter). Results for NO_2_ assimilation show very little improvement, and the VCL sensitivity is low (consequently further results not shown in this paper). It was also found that PM_2.5_, especially sulfate and crustal materials, significantly improve AQ and AQHI forecasts. For other sub-species of PM_2.5_, lesser impact was found. Moreover, when all sub-species are assimilated altogether, the overall improvement does not increase significantly as compared to the sulfate and crustal material assimilation experiment, i.e. there is no cumulative impact or negative synergy found among other PM_2.5_ sub-species; sulfate and crustal assimilation has the most impact within the PM_2.5_ family. The success of assimilation for a particular species seems to be linked with the intrinsic lifetime of the species and the season. For example, shorter lifetime pollutants (nitrogen oxides) have less impact on the AQ 48-h forecast than longer lifetime species (i.e. sulfate or crustal material) which is due to the fact that, given a short lifetime, the information of assimilation is not transported over large regions. Surface ozone has less impact on surface data assimilation than PM_2.5_ in summer since the former is strongly related to photochemistry and diurnal cycles as compared to the latter so that the memory of assimilation is lost more rapidly with ozone than that for PM_2.5_. Finally, whenever the impact of assimilation on AQ forecasts is significant, getting the right vertical correlation (VCL) is critical to its success. The optimum vertical correlation length for assimilation is related to the lifetime of the species but also to the structure of the boundary layer. In winter, the vertical correlation length is about half of what it is in summer which is consistent with lower boundary layer depth during that season. The memory of assimilation is limited by atmospheric lifetime so that an inverse relationship is likely to take place between the optimum VCL and the pollutant’s lifetime. In winter, results are similar to summer except that the optimum vertical correlation is weaker because boundary layer depth is lower and consequently the impact of assimilation is less obvious.

As a final summary, we can state the following:

1. This paper presents for the first time the results of surface data assimilation of chemical species using a Canadian air quality model (i.e. GEM-MACH). Rarely, in the literature, O_3_, PM_2.5_, and NO_2_ have been assimilated together in order to assess the combined impact of the three pollutants (through AQHI in here).
2. Ozone and PM_2.5_ assimilation has a statistically significant impact (*p* < 0.05) on model air quality and AQHI forecasts for almost the whole 48-h forecast period in any season.
3. Assimilating the three pollutants together (PM_2.5_, ozone, and NO_2_) has a positive impact on the AQHI forecast which extends beyond 48 h and improves the AQ forecasts in both seasons at a majority of sites in North America. An absence of cumulative impact or positive synergy in the computation of the AQHI using these three pollutants was observed. This is believed to be due to the efficient control of the biases of the input objective analysis (see Robichaud et al. 2016).
4. Results obtained through sensitivity tests efficiently determine the optimum vertical correlation length (VCL) for assimilation. The method used shows an efficient way to tune VCL before performing assimilation using real-world data.
5. Lifetime and VCL is related to the memory of assimilation with less impact for NO_2_ (less than a few hours) and ozone (half a day in summer to few days in winter) and the greatest impact to sulfate and crustal material (beyond 48 h for PM_2.5_). We suggest that the period over which there is a significant improvement due to assimilation could be an alternate measure of the pollutant atmospheric lifetime.

Improvement of air quality forecasting depends on how objective analysis is used to re-initialize the model. In particular, getting the right VCL is critical. On the other hand, better COAs need to be developed which include a better theory on how to characterize covariance error statistics, horizontal correlation length (Ménard et al. 2016), and better surface observation operators related to land use (i.e. vector H in Eq. 1) (see Bédard et al. 2015). Future work will incorporate online assimilation based on results obtained here. Finally, we suggest that FC2 should be dropped as a metric for ozone surface chemical data assimilation not being. However, FC2 turns out to be quite sensitive for estimating the performance of PM_2.5_.

### Electronic supplementary material

Fig. S1 Impact of assimilation of PM_2.5_ (units in μg/m3) on the 48-h air quality forecasts (winter case, i.e. January 2014) on A) mean absolute bias, B) standard deviation of OmP and mean bias, C) FC2. (GIF 16 kb)

High Resolution Image (EPS 1890 kb)

Fig. S1B (GIF 21 kb)

High Resolution Image (EPS 1939 kb)

Fig. S1C (GIF 15 kb)

High Resolution Image (EPS 1834 kb)

Fig. S2 Impact of assimilation of ozone (units in ppbv) on the 48-h air quality forecasts (summer case, i.e. July 2012) on A) mean absolute bias, B) standard deviation of OmP and mean bias, C) FC2. (GIF 18 kb)

High Resolution Image (EPS 1917 kb)

Fig. S2B (GIF 23 kb)

High Resolution Image (EPS 1964 kb)

Fig. S2C (GIF 16 kb)

High Resolution Image (EPS 1731 kb)

Fig. S3 Impact of combining assimilation of the three pollutants (PM2.5, ozone and NO2) on the AQHI performance (00Z case). Total error for A) July 2012 B) January 2014. (GIF 17 kb)

High Resolution Image (EPS 1714 kb)

Fig. S3B (GIF 18 kb)

High Resolution Image (EPS 1729 kb)

Fig. S4 Percentage improvement of mean absolute bias (based on 24 h forecast in North America) (winter case i.e. Januray 2014), A) PM_2.5_ B) Ozone. (GIF 40 kb)

High Resolution Image (EPS 1714 kb)

Fig. S4B (GIF 42 kb)

High Resolution Image (EPS 1605 kb)

## Appendix 1. Definition of metrics

Given **O** = {*O*_*i*_} and **X** = {*P*_*i*_}, respectively the observed pollutant concentrations and the interpolated model’s prediction *P* at the point of observation of an ensemble of measurements stations *N*, *i* = 1,2,...,*N*, the following metrics are defined:

- Mean bias (*B*):

A.1 $$ B=\frac{1}{N}\sum_{i=1}^N\left({O}_i-{P}_i\right) $$

In the text, the bias (*B*) is presented as Mean (OmP) for model prediction.

- Absolute bias (*AB*):

A.2 $$ AB=\frac{1}{N}\sum_{i=1}^N\mathrm{ABS}\left({O}_i-{P}_i\right) $$

Absolute bias is similar to the bias computation except that the absolute value is taken from the difference between O and P.

- Unbiased root mean square error (URMSE) (i.e., standard deviation of O-P):

A.3 $$ \mathrm{Std}.\mathrm{dev}.=\sqrt{\frac{1}{N-1}\sum_{i=1}^N{\left\{\left({O}_i-{P}_i\right)-\left(\overline{ O i- Pi}\right)\right\}}^2} $$

In the text, the RMSE is presented as std. dev. (OmP) since for large *N*, both unbiased RMSE and standard deviation formulation are equivalent.

- FC2 (frequency of value within a factor 2 compared to observations):

A.4 $$ FC2=\frac{H}{N}\times 100 $$

where *H* is the count when the ratio O_i_/X_i_ is within the range 0.5 et 2, and *N* is the number of total observations used in the analysis. Note that Eqs. A.1, A.2, and A.3 respectively evaluate the systematic bias, the random error, and the reliability. Validation is performed at each hour (00Z to 48Z) unless stated otherwise.

- Total error

A combined index can be used to summarize the performance expressed by both bias and random error. That is the total error, TE, defined as below:

A.5 $$ TE=\sqrt{\left({AB}^{\ast } AB+\mathrm{STD}.\mathrm{DEV}.{}^{\ast}\mathrm{STD}.\mathrm{DEV}\right)} $$

where AB and STD.DEV. are respectively given in Eqs. A.2 and A.3.
