# Supplementary figures and images for: Surface data assimilation of chemical compounds over North America and its impact on air quality and Air Quality Health Index (AQHI) forecasts
Source: Air Qual Atmos Health. 2017 Jun 10;10(8):955–70. doi: 10.1007/s11869-017-0485-9 (PMC5660843; doi:10.1007/s11869-017-0485-9)

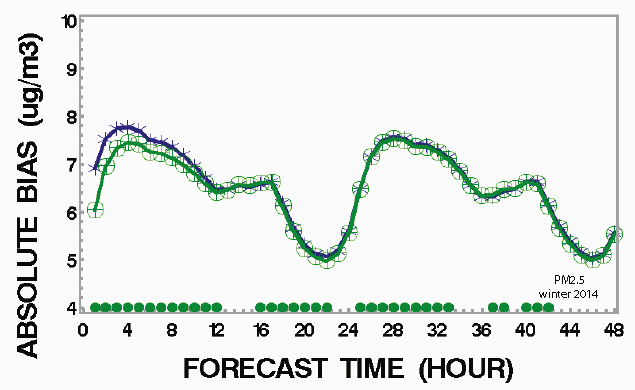

Supplement: Supplementary file 1 — Impact of assimilation of PM2.5 (units in μg/m3) on the 48-h air quality forecasts (winter case, i.e. January 2014) on A) mean absolute bias, B) standard deviation of OmP and mean bias, C) FC2. (GIF 16 kb) [file 11869_2017_485_Fig8_ESM.gif]

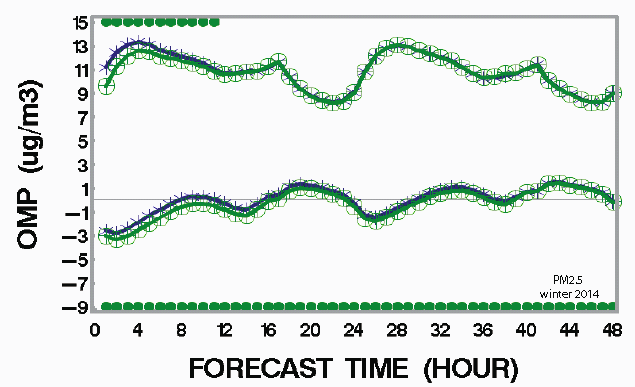

Supplement: Supplementary file 3 — (GIF 21 kb) [file 11869_2017_485_Fig9_ESM.gif]

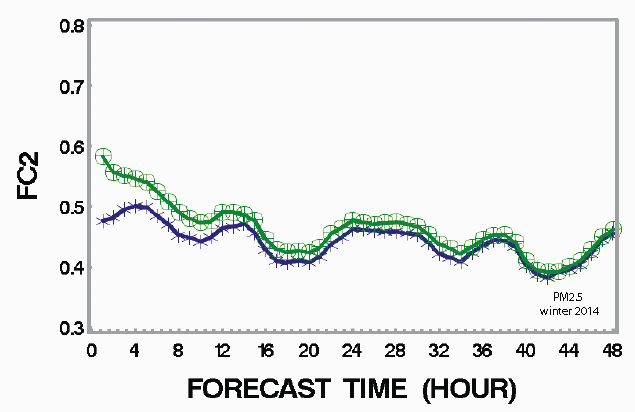

Supplement: Supplementary file 5 — (GIF 15 kb) [file 11869_2017_485_Fig10_ESM.gif]

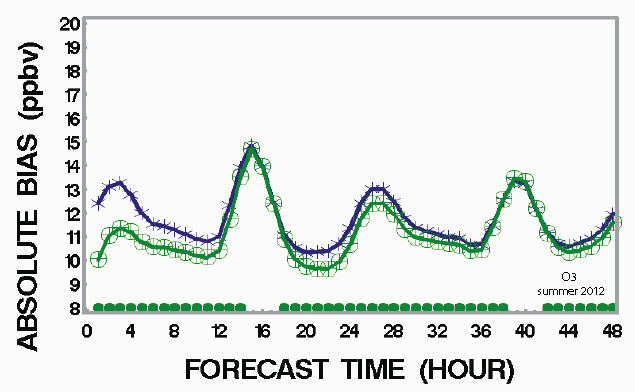

Supplement: Supplementary file 7 — Impact of assimilation of ozone (units in ppbv) on the 48-h air quality forecasts (summer case, i.e. July 2012) on A) mean absolute bias, B) standard deviation of OmP and mean bias, C) FC2. (GIF 18 kb) [file 11869_2017_485_Fig11_ESM.gif]

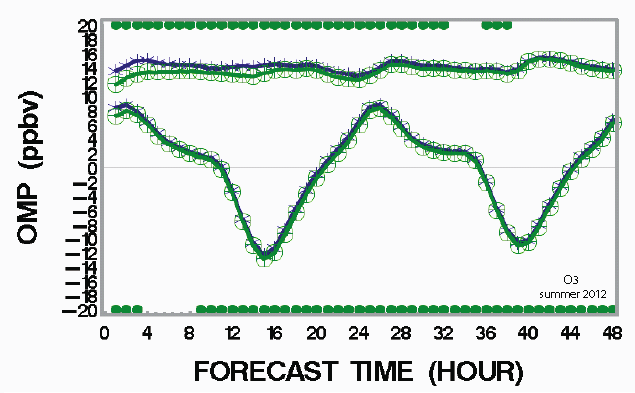

Supplement: Supplementary file 9 — (GIF 23 kb) [file 11869_2017_485_Fig12_ESM.gif]

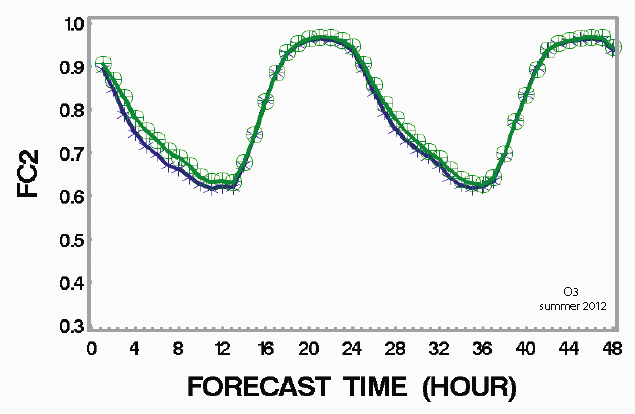

Supplement: Supplementary file 11 — (GIF 16 kb) [file 11869_2017_485_Fig13_ESM.gif]

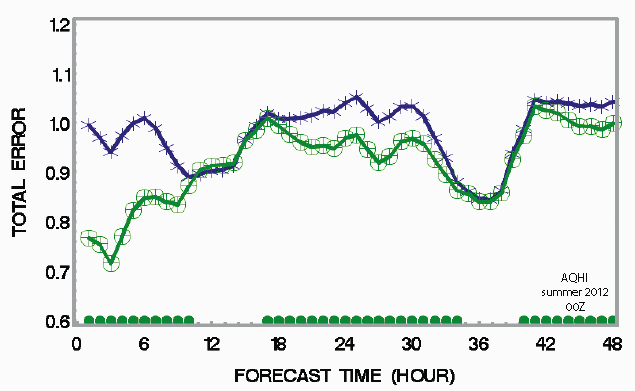

Supplement: Supplementary file 13 — Impact of combining assimilation of the three pollutants (PM2.5, ozone and NO2) on the AQHI performance (00Z case). Total error for A) July 2012 B) January 2014. (GIF 17 kb) [file 11869_2017_485_Fig14_ESM.gif]

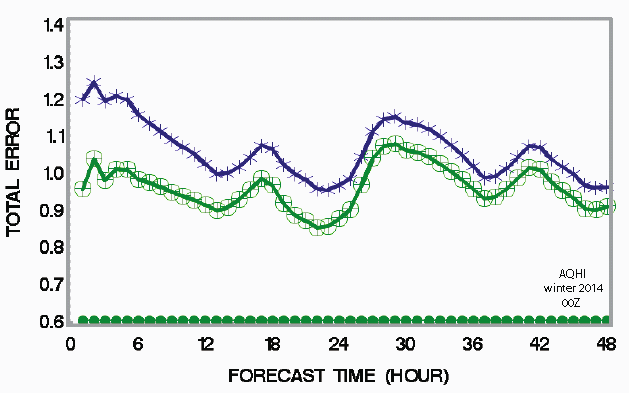

Supplement: Supplementary file 15 — (GIF 18 kb) [file 11869_2017_485_Fig15_ESM.gif]

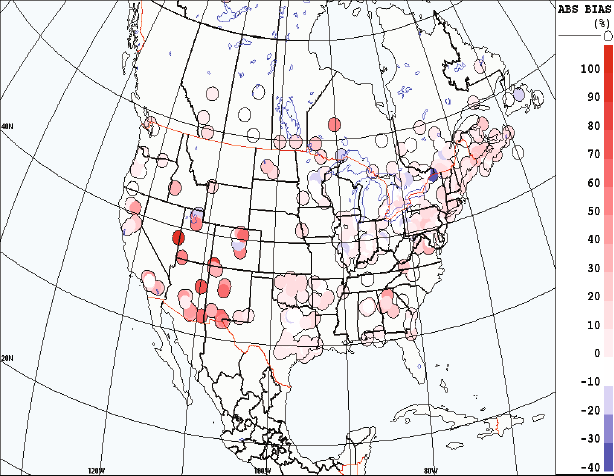

Supplement: Supplementary file 17 — Percentage improvement of mean absolute bias (based on 24 h forecast in North America) (winter case i.e. Januray 2014), A) PM2.5 B) Ozone. (GIF 40 kb) [file 11869_2017_485_Fig16_ESM.gif]

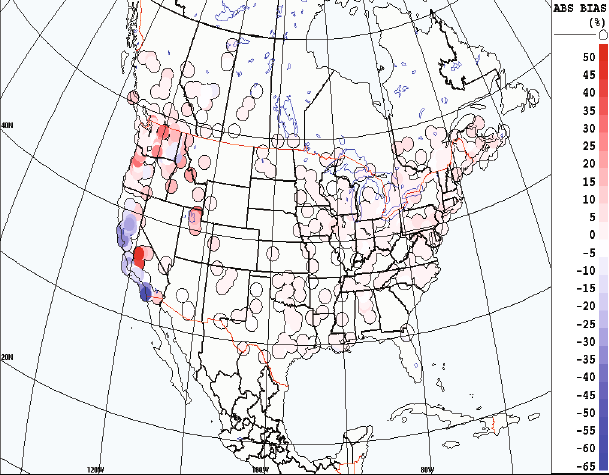

Supplement: Supplementary file 19 — (GIF 42 kb) [file 11869_2017_485_Fig17_ESM.gif]
